# Special Issue “Control of Postharvest Pathogenic *Penicillium*”

**DOI:** 10.3390/jof8090947

**Published:** 2022-09-09

**Authors:** Paloma Sánchez-Torres, Mónica Gandía

**Affiliations:** 1 Instituto de Biología Molecular y Celular de Plantas (IBMCP-UPV), Ingeniero Fausto Elio s/n, 46022 Valencia, Spain; 2 Food Technology Area, Preventive Medicine and Public Health, Food Science, Toxicology and Forensic Medicine Department, Universitat de València, Vicente Andrés Estellés s/n, 46100 Valencia, Spain

Postharvest diseases cause high economic losses in the global citrus and pome fruit industry. The fungal genus *Penicillium* produces the most economically important postharvest diseases of fresh citrus and pome fruits worldwide. Within this genus, it is worth highlighting *Penicillium digitatum*, *Penicillium italicum* and *Penicillium expansum*, which affect fruit quality, reduce the consumption of fresh fruit and contribute significantly to food loss. Some of them also produce mycotoxins with negative consequences to human health. Control of these pathogens is carried out by fungicides; however, the appearance of fungicide-resistant species makes disease control difficult, which results in concern and increases the need for new compounds and target discovery. Therefore, new approaches and tools are required to combat *Penicillium* pathogens during storage.

A complex interplay exists between antifungal resistance and virulence expressed by pathogenic fungi. Currently marketed antifungals are limited, when compared to antibacterial compounds. Comparative genomic and transcriptomic studies have indicated several new potential antifungal targets, which are currently under analysis. Among those, factors involving virulence and pathogenesis could provide new insights for the development of novel compounds, such as antifungal proteins or peptides.

This Special Issue focuses on different approaches developed to control pathogenic *Penicillium* species during postharvest to avoid antifungal drug resistance mechanisms and on potential new target strategies to control fungal infections based on virulence factors and signal transduction pathways underlying the control of infection mechanisms.

A total of eight articles give potential alternatives to the use of fungicides to control these postharvest diseases and fungal growth, including biological control using other microorganisms, use of essential oils, antifungal proteins or regulation of specific genes that can be crucial in virulence, fungal growth or other biological aspects of fungi.

One of these papers, authored by OuYang and co-workers [1], examines the mechanism of citronellal, a typical terpenoid of *Cymbopogon nardus* essential oil. This compound exhibits its antifungal activity against *P. digitatum* by reducing the levels of ergosterol, an important compound of the fungal cell membrane, to maintain the integrity and fluidity. The study provides new knowledge about the antifungal mechanism of citronellal, pointing to the *ERG3* gene as the key regulatory site in response to citronellal treatment.

The regulation of specific genes can contribute to the fungal growth, virulence or pathogenesis development. To advance in the control of postharvest fungal diseases, it is important to explore the mechanism that fungi use in the infection processes. Three of the articles published in this Special Issue follow these lines. Xu et al. [2] studied the role of arginine methyltransferase proteins (PRMTs), which modulate many cellular processes. In the pome pathogen *P. expansum*, they identified four *PRMT* genes and the elimination of one of them (*PeRmtC*) affects the development, pathogenicity and secondary metabolism of the fungus, regulating conidiation via controlling genes involved in this pathway. Deletion mutants exhibited a reduction in conidia production and a delay in conidia germination, affecting fungal virulence. On the other hand, de Ramón-Carbonell and Sánchez-Torres [3] examined Zn_2_Cys_6_ transcription factors in *P. digitatum* and demonstrated an indirect role of one of these genes, *PdMut3*, involved in cell integrity. Deletion mutants were affected in fungal growth, conidiophore development or hypha morphology, showing alterations in cell wall and chitin content. *PdMut3* could be related to metabolism through peroxisomes development, regulating their degradation and could also be negatively controlled by the *PdSte12* transcription factor involved in Fus3 MAPK metabolic pathway. These results point to this gene as a potential target for the development of new antifungal compounds.

Finally, Li and co-workers [4] analyzed Calcium (Ca2+)/calmodulin-dependent protein kinases (CaMKs), identifying a new gene in *P. italicum* (*PiCaMK1*). Results obtained in this work suggest that *PiCaMK1* function involves the regulation of multiple physical and cellular processes of this pathogen, including growth, conidiation, virulence, and environmental stress tolerance.

On the other hand, the many problems associated with synthetic fungicides forces us to look for more natural, sustainable compounds with a minimal impact on the environment. Gandía and colleagues [5] describe the potential use of antifungal proteins (AFPs) as alternative tools in the control of postharvest diseases. AFPs secreted by filamentous ascomycetes have great potential as new biofungicides due to their characteristics. The authors evaluated the potential application of different AFPs obtained from different fungi: PAFB and PAFC from *Penicillium chrysogenum* [6,7] and NFAP2 from *Neosartorya fischeri*, [8] compared to one of the first described AFPs (PAF from *P. chrysogenum*) [9] and the highly active PdAfpB from *P. digitatum* [10] and PeAfpA from *P. expansum* [11]. In vitro studies were performed with these different proteins. In vivo assays with orange and apple fruits showed a delay in the *P. digitatum* and *P. italicum* infection of orange fruits and the same result in the *P. expansum* infection of apples when the fruits were treated with PAFB. Antifungal potential to control postharvest diseases in the case of PeAfpA had been demonstrated in previous works in orange and apple fruits too [11,12]. All of these results support the employment of AFPs as putative antifungal compounds in the control of fungal postharvest diseases.

Last but not least, biocontrol is another option in the control treatment of these fungal diseases. The application of biological control agents present in the surfaces of vegetables and fruits is also a very appropriate alternative method to synthetic fungicides. In this sense, Dor and colleagues [13] present quorum-sensing signaling molecules (QSMs) secreted by bacteria as a method to control fungal pathogenicity. They demonstrated that a bacterial N-acyl homoserine lactonase can also efficiently degrade patulin, a fungal mycotoxin secreted by *P. expansum,* and that it could have a potential use in postharvest disease treatments. Moreover, yeast and bacteria can be used in biocontrol to preserve food. The use of edible packaging films is becoming a strategy widely employed by the food industry. García-Bramasco et al. [14] evaluated the addition of *Debaryomyces hansenii* yeast in chitosan films for controlling *P. italicum*. The incorporation of antagonistic yeast improved the mechanical resistance of the films and these could be used for citrus packaging in the future. Finally, Hammami and coworkers [15] carried out a screening of different yeasts and bacteria isolated from the peel of citrus fruits and analyzed their antagonistic effect against *P. digitatum* and *P. italicum*. Two different yeasts (*Candida oleophila* and *D. hansenii*) and three bacteria isolated (*Bacillus amyloliquefaciens*, *B. pumilus* and *B. subtilis*) reduced the incidence of decay in orange and lemon fruits by *P. digitatum* and *P. italicum*, confirming their potential use as biocontrol agents in postharvest control.

To summarize, the articles included in this Special Issue cover different and alternative treatments to control fungal postharvest diseases. The works have been carried out using different pathogens inside the *Penicillium* genus and help to increase knowledge in this field.

We hope that this Special Issue has contributed to further research into new alternatives for the control of *Penicillium* diseases. We highly appreciate the contributions of each of the authors, making this Special Issue possible and thank all of them for sharing their expertise. We express our gratitude to all the reviewers who contributed, and, furthermore, we are grateful to the “Journal of Fungi” for their valuable support and for giving us this opportunity to provide advances in the field of “Control of Postharvest Pathogenic *Penicillium*”.
